# Alternative pathway activation in pregnancy, a measured amount “complements” a successful pregnancy, too much results in adverse events

**DOI:** 10.1111/imr.13169

**Published:** 2022-11-15

**Authors:** Kate Smith‐Jackson, Richard Alexander Harrison

**Affiliations:** ^1^ Complement Therapeutics Research Group, Translational and Clinical Research Institute, Faculty of Medical Science Newcastle University Newcastle‐upon‐Tyne UK; ^2^ The National Renal Complement Therapeutics Centre (NRCTC) Newcastle‐upon‐Tyne Hospitals NHS Foundation Trust Newcastle‐upon‐Tyne UK; ^3^ Institute of Infection and Immunity, School of Medicine Cardiff University Cardiff UK

**Keywords:** complement, obesity, pre‐eclampsia, pregnancy

## Abstract

During pregnancy, the maternal host must adapt in order to enable growth of the fetus. These changes affect all organ systems and are designed both to protect the fetus and to minimize risk to the mother. One of the most prominent adaptations involves the immune system. The semi‐allogenic fetoplacental unit has non‐self components and must be protected against attack from the host. This requires both attenuation of adaptive immunity and protection from innate immune defense mechanisms. One of the key innate immune players is complement, and it is important that the fetoplacental unit is not identified as non‐self and subjected to complement attack. Adaptation of the complement response must, however, be managed in such a way that maternal protection against infection is not compromised. As the complement system also plays a significant facilitating role in many of the stages of a normal pregnancy, it is also important that any necessary adaptation to accommodate the semi‐allogenic aspects of the fetoplacental unit does not compromise this. In this review, both the physiological role of the alternative pathway of complement in facilitating a normal pregnancy, and its detrimental participation in pregnancy‐specific disorders, are discussed.

**Abbreviations**
aHUS: atypical hemolytic uraemic syndrome
AP: alternative pathway
aPL: antiphospholipid antibody
APS: antiphosphospholipid syndrome
AR: acrosome reaction
AT1 receptor: angiotensin II type 1 receptor
BMVECs: brain microvascular endothelial cells
BPH/5: blood pressure high/5 mouse
CAPS: catastrophic antiphoslipid syndrome
CD46: membrane cofactor protein
CD55: decay accelerating factor
CMAS: CMP‐sialic acid synthase
CP: classical pathway
CR2: complement receptor 2
CR3: complement receptor 3
DMARD: disease‐modifying anti‐rheumatic drug
dpc: days postcoitus
DSC: decidual stromal cells
ESRD: end‐stage renal disease
EVT: extravillous trophoblast cells
FB: factor B
FD: factor D
FH: factor H
FI: factor I
GDM: gestational diabetes
GMVECs: glomerular vascular endothelial cells
hCG: human chorionic gonadotrophin
HCQ: hydroxychloroquine
HELLP: hemolysis, elevated liver enzymes and low platelet syndrome
HNF: hepatocyte nuclear transcription factors
IAI: intra‐amniotic infection
IDO: indoleamine‐2‐3‐dioxygenase
IUGR: intrauterine growth restriction
LP: lectin pathway
LPS: low‐dose endotoxin
MAC: membrane attack complex
MAP: mean arterial pressure
MCP: membrane cofactor protein
MHC: major histocompatibility complex
MMP‐9: matrix‐metalloproteinase 9
NICE: National Institute for Health and Care Excellence
p‐aHUS: pregnancy‐associated atypical hemolytic uraemic syndrome
PEARS: pregnancy exercise and nutrition research study
PIGF: placental growth factor
PNH: paroxysmal nocturnal hemoglobinuria
PROMISSE: predictors of pregnancy outcome biomarkers in antiphospholipid antibody syndrome and systemic lupus erythematosus
PTB: preterm birth
RU486: progesterone antagonist
sCR1: soluble complement receptor 1
SCRs: short consensus repeats
sFLT‐1: soluble fms‐like tyrosine kinase 1
SGA: small for gestational age
SLE: systemic lupus erythematosus
SNP: Single nucleotide polymorphisms
T2DM: type 2 diabetes mellitus
TMA: thrombotic microangiopathy
TNF‐α: tumor necrosis factor alpha
TP: terminal pathway
uNK: uterine natural killer cells
VEGF: vascular endothelial growth factor
WAT: white adipose tissue
ZP: zona pellucida

## 1 INTRODUCTION

Pregnancy leads to profound maternal physiological and metabolic changes. These are required to accommodate and nurture the developing fetus, and affect all organ systems.^1^ In a normal pregnancy, these orchestrated multisystem changes enable successful delivery of a healthy baby and minimize the risks associated with pregnancy to the mother. However, pregnant women with pre‐existing health conditions are at increased risk of adverse events in pregnancy. These can arise either indirectly, following decompensation of their underlying disease, or directly, from a collection of pregnancy‐specific disorders; women with no underlying premorbidity are at risk of developing pregnancy‐specific disorders due to the “stress” test of pregnancy.^2^ The effects of adverse events in the mother are not limited to the pregnancy, they impact upon a woman's risk of developing disease in later life. Similarly, infants that are exposed to adverse events in utero have increased risk of developing vascular dysfunction in later life. Maternal morbidity and mortality remain a significant healthcare concern across the globe^3^; thus, understanding, identification, and treatment of adverse events in pregnancy is of vital public health importance.

The immune system undergoes profound changes in order to tolerate the semi‐allogeneic feto‐placental unit. These changes are required both for engraftment and for the fetus to thrive within the maternal environment. Adaptations in the immune system prevent rejection of the engrafted unit while simultaneously protecting both mother and fetus from microbial attack. Maternal immune tolerance is achieved through several adapted immune mechanisms. For example, attenuation of adaptive immunity through the generation of an immunologically privileged environment within the maternal decidua prevents access of maternal T‐ and B cells to fetal tissue.^4^ The villous trophoblasts, which encase the placental villi providing nutrient exchange, lack major histocompatibility (MHC) molecules, enabling them to escape immunological detection.^5^ The extravillous trophoblast cells (EVT), which invade the uterine lining, express unique MHC molecules; these limit their recognition by T cells, but still enable them to act as major ligands for natural killer (NK) cells and macrophages.^5^ Uterine NK cells (uNK) and macrophages are the predominant immune cell populations within the decidua and myometrium, and participate in trophoblast invasion through pro‐angiogenic activity.^4^, ^6^, ^7^ Additionally, the placenta expresses high levels of indoleamine 2,3‐dioxygenase (IDO). IDO activity protects the developing fetus from maternal T cell responses.^8^ Together, these measures provide an effective modification of adaptive immunity within the fetoplacental environment such that both the host and the fetus remain protected.

The situation with respect to complement is different. The complement system plays an integral role in normal pregnancy, from fertilization to parturition. Any adaptation to prevent identification of the fetoplacental unit, and the fetus, as non‐self, must simultaneously permit complement to play its essential role in normal pregnancy and to continue to provide host defense against infection.

Women with active autoimmune conditions are more vulnerable to adverse events in pregnancy,^9^ and the fine balance that the immune system has to maintain in pregnancy is illustrated by disorders in which it is sensitized. This is evidenced, for example, in pre‐eclampsia. Pre‐eclampsia is a multisystem disorder classically defined by new onset of hypertension coupled with the presence of additional endothelial dysfunction, and carries significant risk not just for the mother, both during pregnancy and in later life, but also for the fetus.^10^ The incidence of pre‐eclampsia is increased in women with risk factors for chronic inflammation (eg obesity), also with a preponderance for women with autoimmune diseases, such as antiphospholipid syndrome (APS) and systemic lupus erythematosus (SLE), and dysregulated complement activation appears to provide a major effector mechanism.

While there are many immune‐related mechanisms at play in both normal pregnancy and in pregnancy‐associated disorders, in this review, we confine ourselves primarily to discussion of the role of the AP in facilitating a normal pregnancy, and its participation in the inflammatory‐mediated injury of pregnancy‐specific disorders.

## 2 THE ROLE OF COMPLEMENT ACTIVATION IN NORMAL PREGNANCY

### 2.1 Conception

It is quite clear that complement does not play an essential role in conception; mice deficient in complement components, particularly, in the context of this review, C3 and Factor B (FB), remain fertile. However, there are considerable data that support a facilitatory role for components of the system. During conception, capacitated spermatozoa bind to the zona pellucida (ZP) of the oocyte and trigger the acrosome reaction (AR). This exocytosis event releases the acrosomal contents and exposes membrane cofactor protein (MCP, CD46), located on the inner acrosomal membrane of the sperm, to the reproductive tract environment.^11^ While CD46 exists as multiple isoforms, with individual isoform expression determined by the tissue or cell type, it should be noted that the isoform of CD46 expressed on the acrosomal membrane is unique to spermatozoa, suggestive of a highly specific role.^12^, ^13^

It has been shown that acrosin, an acrosomal protease released during the AR, can cleave C3 to generate a C3b‐like molecule. This nascent C3b has the transient ability to bind covalently to adjacent surfaces, including both the acrosomal and the ovum plasma membranes. Subsequent interactions, either between C3b bound to the ovum and CD46 expressed on the spermatozoa, or between C3b and/or its degradation product iC3b and complement receptors (CR1, CD35; CR3, CD11b/CD18) on the ovum, has been proposed to facilitate fusion of the sperm with the oocyte.^11^ In the same study, it was noted that subsaturating concentrations of C3b promote penetration and that saturating C3b inhibits these interactions. One possible rationale for this is that dead or damaged spermatozoa are more strongly complement‐activating than healthy spermatozoa, and therefore more likely to produce saturating amounts of C3b, thus enhancing fertilization by healthy spermatozoa.

A more recent study confirmed, in part, these observations, and supported the facilitated fusion hypothesis.^14^ In this study, using sera depleted of specific components, it was shown that C3 activation could be initiated in a CRP‐C1q‐C2‐dependent pathway, and that activation was amplified through the C3 feedback loop. C3b deposited on the inner acrosomal membrane could be converted to iC3b by Factor I (FI) using Factor H (FH), but not MCP, as a cofactor. Interestingly, activation of the terminal pathway (TP) was not seen. This strengthens the hypothesis that iC3b can act as a ligand to facilitate fertilization through enhancing sperm–egg interactions. In their conclusion, the authors suggested that complement activation is precise and measured when compared to non‐specific activation by foreign antigens, with fragment deposition targeted to a precise cellular location (the acrosomal inner membrane), and tightly controlled, such that a restricted amount of C3b/iC3b is deposited.^14^

### 2.2 Pre‐implantation

Once fertilized, and prior to implantation of the blastocyst in the maternal decidua of the uterus, the rapidly dividing zygote migrates down the fallopian tube and into the uterus. Mucosal secretions in the fallopian tube and uterus contain all of the components required to mount a complement attack.^15^ Thus, during this passage, which takes around 9 days in humans, the semi‐allogeneic fertilized egg or blastocyst may be subject to complement attack. A study using cryopreserved human embryos has shown that mucosal complement can target healthy embryos prior to implantation, evidenced by deposition of C1q and activation fragments of C3 (C3b/iC3b and C3d) on the blastomere membrane.^16^ The embryos also expressed the membrane‐bound regulators CD46, CD55 and CD59 on their surface, and recruited the soluble regulators FH and C4bp. Efficient protection of the embryo surface was demonstrated both by the absence of C5 on blastomere membranes (C5 deposition was confined to the ZP, the extracellular matrix that surrounds the blastomere membrane) and the observation that C3 fragment deposition was primarily as C3d, the inactive limit degradation product of surface‐bound C3b.^16^ Interestingly, C5 deposition was identified in the ZP but not on the blastomere membrane. The ZP also contained FH but not membrane‐bound regulators or C4bp. This suggests that the ZP may be a site of concentrated complement attack, with AP activation/amplification playing a prominent part. Based on these observations, the authors hypothesized that one role of the ZP might be to protect the inner embryo from complement attack, providing a protective shield that diverts activation products away from the blastomere membrane.

This protection must be achieved without compromising pre‐implantation pregnancy‐specific roles of complement. C3 is synthesized and secreted into the reproductive tract by the embryo, oviduct epithelial cells, and the endometrium. The rate of synthesis follows a cyclical pattern, with highest production during estrus.^17^, ^18^, ^19^ Activation of this to C3b, and the subsequent FI‐mediated breakdown of this to iC3b, plays an embryotrophic role as the embryo develops, presumably through an interaction with CR3, which is expressed by the embryo, with iC3b the most potent embryotrophic ligand.^20^, ^21^ This in vitro work is further validated in mice that are deficient in C3. These have smaller blastocytes, suggesting a growth compromise in the absence of C3.^21^, ^22^

Additionally, complement plays a role in the successful decidualization of the endometrium prior to implantation. Decidualization results in the transformation of uterine stromal cells into decidual stromal cells (DSC). DSC possess unique properties that induce immunological, hormonal, and vascular changes, creating a maternal decidual layer that enables the blastocyte to implant, and the trophoblasts to invade, and leading ultimately to the successful establishment of a functioning placenta.^23^, ^24^ Studies in non‐human primates treated with human chorionic gonadotrophin (hCG) showed upregulation of endometrial expression of C3, with a specific increase identified within the uterine stromal tissue. Similar results were obtained in human endometrial tissue in response to hCG, with an increase in C3 mRNA and protein expression, again confined to the stromal tissue. Thus, the embryo, through its release of hCG, is able to communicate and signal to the endometrium, leading to an upregulation of C3 within the uterine stromal compartment, suggesting a role for C3 within the decidual immune environment. An increase in complement activation is also met with an increase in CD55 (decay accelerating factor; DAF) expression within the uterine luminal epithelium, underlying the need for balanced control of complement activation during decidualization.^25^, ^26^

C1q is expressed in the decidual endothelial cells and synthesized locally by EVT. Specifically during pregnancy, C1q acts in a physiological manner to support the adhesion of the endovascular trophoblasts to the uterine endothelium during the remodeling of the spiral arteries. Additionally, EVT secrete C1q that binds to extracellular matrix proteins within the decidua, which the EVT use to further their invasion of the decidua. In vivo studies using the C1q deficient pregnant mice have shown an impaired labyrinth development and vessel remodeling, illustrating, the importance presence of C1q for the successful formation of the murine placenta.^27^, ^28^

These essential roles of an intact complement system in the reproductive tract in early pregnancy highlight the stringent need for adaptation of the embryo to survive in an otherwise hostile environment. Without these adaptations, the embryo would not survive, and pregnancy would be terminated. In addition to the complement‐specific processes outlined above, additional protection against an immune attack on the developing embryo may be provided by immunological priming.^29^ Constituents of seminal fluid influence the maternal cellular response; postmating, there is an expansion of lymphocyte populations, with pronounced homing of these to the site of implantation. It has been hypothesized that this may facilitate maternal tolerance of the embryo.

### 2.3 Implantation and placental development

**FIGURE 1:**
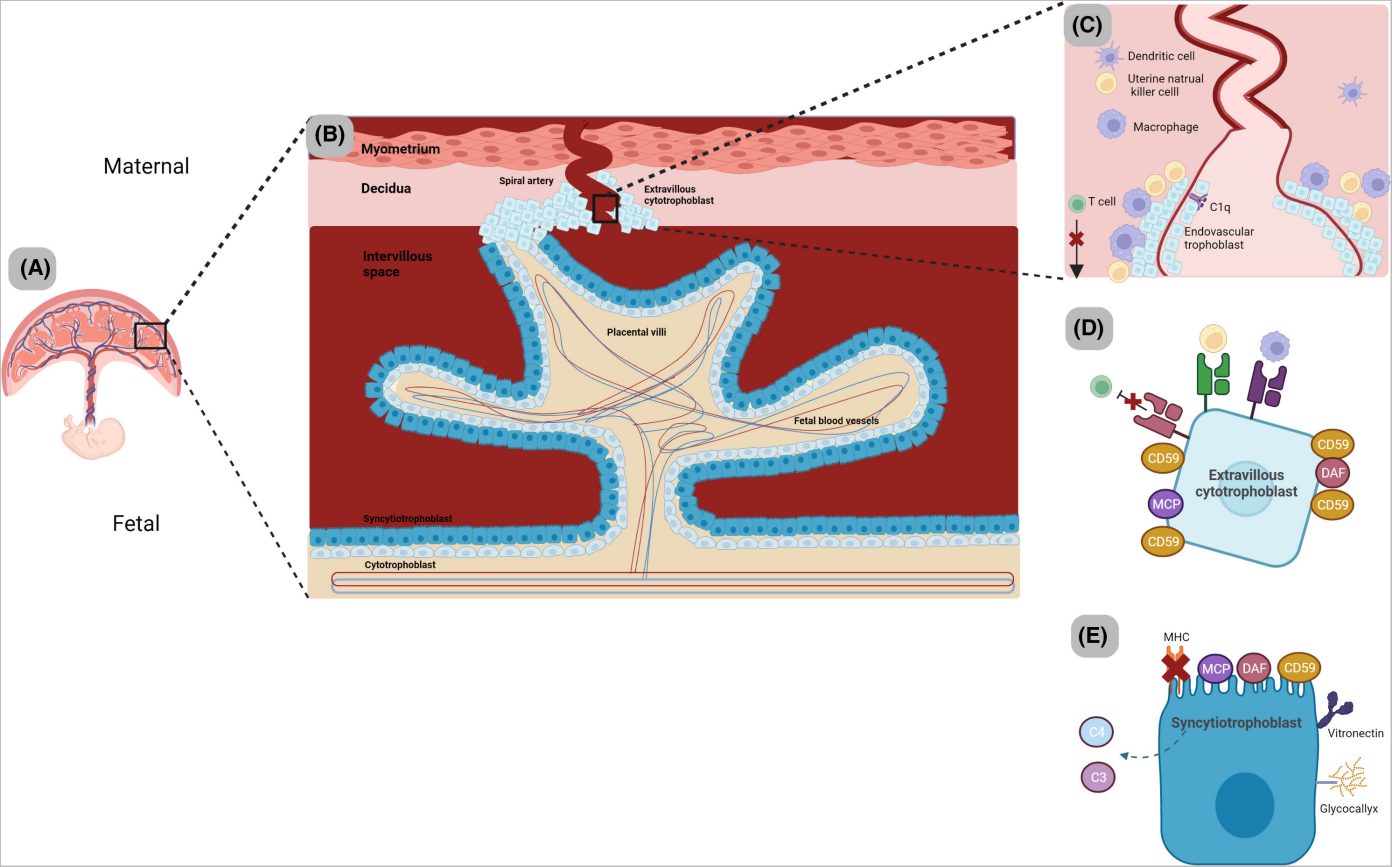
Maternal‐fetal interface. (A) The human hemochorial placenta. (B) A placental villous is formed by a double cell layer of syncytiotrophoblasts (in direct contact with maternal blood) and cytotrophoblasts directly beneath. Fetal blood vessels and fetal macrophages remain housed within the villus. Extravillous trophoblasts (EVTs) depart from the tip of the placental villi and migrate into the maternal decidua, providing anchorage of the villi to the uterine wall. (C) In response, uterine natural killer cells (uNK) are recruited, which promote blastocyte implantation and provide support for the continued EVT migration through the release of cytokines promoting angiogenic activity. The EVT invade the spiral arteries (now termed endovascular trophoblasts), and in union with the uNK remodel the spiral arteries, replacing the vessels inner lining (characterized by loss of smooth muscle cells). This remodeling creates a dilated vessel that has “High flow” and “low resistance” conditions, optimizing blood flow to the placenta. C1q is locally produced from the decidual endothelial cells on the inner side of spiral arteries and from the endovascular trophopblasts. This physiological response appears to provide a functional scaffold between the two cells. The maternal decidua also houses macrophages and T cells. Macrophages restrict T cell activity through the release of IDO and phagocytose apoptotic EVT, as well as contributing to remodeling. (D) The Extravillous trophoblast express unique MHC molecules, which disguises them from T cells, while simultaneously acting as a major ligand for uterine natural killer cells and macrophages. They preferentially express CD59 to protect from lytic attack. (E) The Syncytiotrophoblasts are in direct contact with maternal blood and act as the site of nutrient, gas and waste exchange between the mother and developing fetus. They are immunological undetectable given the absence of any MHC molecules. A thick glycocalyx layer provides further shelter against immunological attack. MCP, DAF, CD59 are all expressed to protect the cell layer from maternal complement attack. Further reinforcement is provided by binding of vitronectin (soluble inhibitor from maternal blood) to prevent C5b‐9 assembly. They have also been shown to be a source of locally produced C3 and C4. Cytotrophoblasts express MCP and CD59 to protect from any complement spill over from the syncytiotrophoblast layer.

**FIGURE 2:**
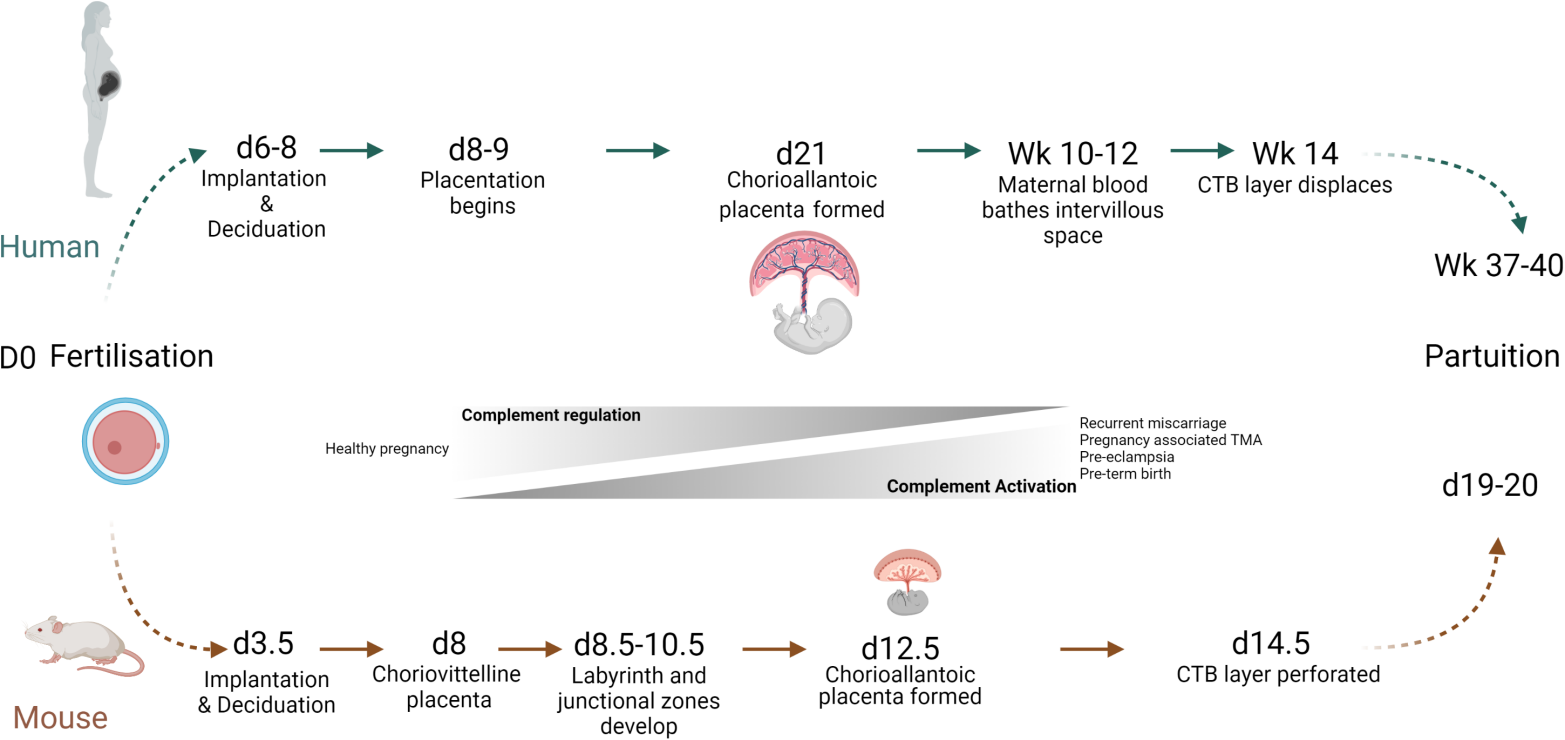
Pregnancy milestones in human and mouse. Humans and mice both form a hemochorial placenta (maternal blood is in direct contact with the chorion). In humans, maternal blood bathes the intervillous space toward the end of the first trimester. In contrast, mice form a primitive placenta (choriovitelline), followed by the definitive (chorioallantoic) placenta around day 11–12.5 postcoitus. In structural contrast to the villous tree formed in the human placenta, mice form a highly interconnected maze‐like structure of chorionic villi which is termed the labyrinth. The labyrinth is supported by the spongiotrophoblast layer (not present in humans). The labyrinth comprises of two layers of syncytiotrophoblasts (SYN) overlaid by a layer of cytotrophobasts (CTB) which directly contact maternal blood. Maternal blood accesses the outermost SYN layer once the CTB layer is perforated (around day 14 postcoitus). Achieving each successive pregnancy milestone requires adequate complement regulation, complement dysregulation and increased activation has been shown to be involved with adverse events in pregnancy.

As the blastocyte progresses along the reproductive tract, the uterine endometrium undergoes a transformation, generating the maternal decidua, the structure in which the blastocyte implants. Extravillous trophoblasts migrate through the maternal deciduas towards the spiral arteries, with extensive remodeling of these transforming them into high flow, low resistance vessels (see Figure 1). This remodeling is necessary to ensure that the growing fetus receives the blood flow and nourishment that it needs (reviewed in^30^). Implantation of the blastocyte into the maternal decidua occurs at around 9 days postcoitus (dpc) for humans and 4 dpc for mice (see Figure 2) and rats.^15^ Both mice and humans form a hemochorial placenta. In this, maternal blood passes through vascular spaces lined with fetal trophoblasts (syncytiotrophoblast) forming the vascular barrier. The syncytiotrophoblast is a multinucleated syncytium with a microvillous brush border that covers the entire length of the chorionic placental villi. The apical membrane is in direct contact with the maternal blood and the basal membrane in contact with the fetal blood vessels; it facilitates transport to the fetus through transcytosis.^31^

Animal models have proved invaluable in developing our understanding of the role of complement in blastocyte implantation and placental development. As stated above, iC3b is embryotrophic and likely contributes to embryo growth and development before the placenta is fully established.^15^, ^22^ Additionally, *C3−/−* mice have a normal number of implantation sites, but an increase in resorptions occurs around 15 dpc, suggesting that C3 is particularly important for placental development rather than implantation.^15^, ^22^ While CD55 and CD46 are heavily expressed in the human placenta, regulating both classical pathway (CP) and AP activation of C3, neither CD55 nor CD46 is expressed in the murine embryo or trophoblast.^32^ In rodents, a novel membrane‐bound protein, Crry, replaces CD55 and CD46 in the placenta.^33^ It regulates deposition of activated C3, combining CD55‐ and CD46‐like regulatory activities,^34^ and is highly expressed on the trophoblast cells of wildtype mice at 7.5 dpc.^35^ Seminal work using the Crry−/− mouse showed the importance of AP regulation for successful maintenance of a normal pregnancy in mice, with Crry knockout leading to embryonic lethality at 10.5 dpc.^35^ In these mice, maximal C3b deposition was seen at 7.5 dpc; this was accompanied by the presence of neutrophils around the trophoblast cells, and preceded death by 3 days. In the same study, the authors further showed that maternal C3 was primarily responsible, with backcrossing onto maternal *C3−/−* mice rescuing the phenotype and preventing the neutrophil influx.^35^ In follow‐up work, the same group demonstrated that backcrossing *Crry−/−* mice onto *C4−/−* mice or B cell deficient mice did not rescue the phenotype, thus neither antibody nor CP activation is involved in embryonic lethality. Similarly, embryonic lethality of *Crry−/−* pups was still observed in the setting of either neutrophil depletion or backcrossing with C5 deficient mice. (While the backcross with C5‐deficient mice did result in rescue of a small minority of pups [5%], a primary dependence of lethality on C3 activation products remains, with C5 activation playing, at most, a minor role.) By contrast, backcrossing the *Crry−/−* mice with *FB−/−* mice rescued the lethality phenotype, with viable *Crry−/−* pups produced. Neither increased C3b deposition nor neutrophil infiltration was observed in this double knockout mice.^36^ From this, the authors concluded that unregulated activation of the AP triggers C3‐dependent neutrophil infiltration of the trophoblast and impaired feto‐maternal tolerance, leading ultimately to fetal demise.^36^ The virtually total dependence of the embryonic lethality phenotype on C3, but not downstream effector arms of complement, suggests that there is either a C3a/C3aR‐ or an iC3b/CR3‐driven, or a mixture of both, mechanism at play. Dual perfusion of the placenta, and thus independence of the fetus on the embryonic yolk sac for nutrition, is achieved in Crry‐sufficient mice at 9.5 dpc. The timing of fetal lethality in *Crry−/−* animals is consistent with a failure in development of the allatonic placental vessels, resulting in impaired vascular supply to the developing fetus at a critical time when its source of nutrients is transitioning from the yolk sac to the placenta.^36^

Work using the *CMAS−/−* mouse has further validated the essential need for complement regulation to enable fetal survival.^37^ Mammalian cells are coated in a dense layer of glycans (the glycocalyx), with sialic acid occupying the outermost position in the bulk of the glycocalyx glycans.^38^ *CMAS−/−* mice lack the sia‐activation enzyme (CMP‐sialic acid synthase [CMAS]), and, as a consequence, the glycocalyx glycans are not sialylated. Sialylation has a major impact on the functioning of glycans, being able to dampen immune responses (reviewed in^39^), and functioning as a ligand for FH and enabling recognition between self and non‐self.^40^ Trophoblasts from wildtype embryos are extensively sialylated. The absence of sialylated glycans following genetic deletion of CMAS resulted in embryonic lethality at 9.5 dpc,^37^ with complement activation on trophoblasts. As with *Crry−/−* mice, complement activation was accompanied by an influx of maternal neutrophils at the fetal‐maternal interface, leading to impaired fetal growth and subsequent demise. Lethality could be rescued by depletion of maternal C3, at 8.5 dpc, through injection of cobra venom factor (a tool compound for systemic C3 depletion). This prevented C3 deposition and infiltration of maternal neutrophils.^37^ Neither C1q nor C4d were detected in immunohistochemical staining of the embryo, indicating that there was no significant role of CP or lectin pathway (LP) in complement activation. By contrast, an increase in properdin staining was seen, consistent with AP activation. While C3a (and C5a) are chemoattractants for neutrophils, suggesting a possible role for neutrophils in lethality, antibody‐induced depletion of neutrophils did not rescue *CMAS−/−* embryos. These data demonstrate that neutrophil recruitment is a secondary effect following excess complement activation by the AP,^37^ and that sialyation is crucial for human trophoblasts protection from maternal complement attack. Recruitment of FH requires the presence of sialylated glycans; thus, in the absence of sialyation, a lack of FH likely contributes to hyperactivation of C3.

In support of this, mutations in FH that affect the sialic acid recognition site have been associated with atypical hemolytic uremic syndrome (aHUS, a rare kidney disease also consequent on dysregulation of the AP),^41^ and genes encoding enzymes involved in sialic acid synthesis and processing have been reported to be differentially regulated in the placenta of women who develop pre‐eclampsia (see later).^42^, ^43^

What is particularly interesting from the above mouse studies is that complement‐dependent embryonic lethality appears to be driven almost entirely by activation (C3a/C3b) and degradation (iC3b) products of C3. Neither neutrophils nor C5 activation products (C5a; C5b‐9, the membrane attack complex of complement [MAC]) appear to play a significant role, and this has implications for possible treatments for recurrent early pregnancy loss. It also appears likely that, in the mouse, surface‐bound FH alone cannot give sufficient protection. While it is not clear why this should be so, one possibility is that FH has a location‐specific role, which is insufficient to protect the fetus.

Collating the above data, derived mainly from pregnancy studies in mouse models, it is clear that, appropriately regulated, the AP plays an integral role in facilitating successful blastocyte implantation within the maternal decidua, in engraftment, and in establishing a functioning placenta. C1q, C4, C5, C6, C9 are all found in the placenta and in the decidual spiral arteries,^44^, ^45^ trophoblast cell lines have been shown to synthesize C3 and C4, and it is logical that the placenta possesses an intact complement system to provide immune defense against pathogens.^46^ In order to protect the allogeneic fetus, activation within the reproductive tract requires tightly regulated control of the system, and the placenta normally achieves this through the high expression of CD55, CD46 and CD59 on EVT throughout the gestation period,^7^ as well as through the recruitment of FH to cell membranes. If regulation fails, the fetus comes under attack from maternal C3, with pregnancy failure the likely outcome.

### 2.4 Circulating complement during pregnancy

Multiple studies have confirmed that pregnant women have an increase in the plasma concentrations of circulating complement proteins and anaphylatoxins when compared to non‐pregnant controls.^47^, ^48^, ^49^

In a recent longitudinal study of complement and complement activation products in pregnant and non‐pregnant women, He et al. evaluated plasma samples taken from the 1st to 3rd trimesters. While <30% of the cohort had three or more samples from which to assess the impact of increased gestation time on complement,^50^ they were able to determine that C3 and C4 increased gradually throughout pregnancy. Levels of C1q, C5a, and sC5b‐9 were similar to non‐pregnant women in the 1st and 2nd trimesters. FB and FH rose during the 1st and 2nd trimesters and then remained stable during the 3rd trimester. An increase in FB seen during the 1st trimester is compatible with enhanced activation potential and the involvement of the AP in placental formation. The concomitant increase in FH levels, and the relative abundance of FH in the 2nd and 3rd trimesters of normal pregnancy, highlight the need for a balanced regulatory response to avoid excessive systemic activation of the AP while, at the same time, permitting local C3‐dependent mechanisms required to maintain a normal pregnancy.

C3a in the 2nd and 3rd trimester, and C5a in 3rd trimester, were higher in pregnant women, with sC5b‐9 remaining unchanged. Collectively, and primarily assessed through the presence of the anaphylatoxins in the maternal circulation, this illustrates that there is systemic activation of the complement system in the 2nd and 3rd trimesters of normal pregnancy. (Given the concentrations of complement proteins in plasma, anaphylatoxin levels are likely to give a more accurate assessment of activation than variations in native protein concentrations.) Comparison of C3a, C4a, and C5a levels were indicative that not all C3a was generated after C4 activation; thus, the AP was likely to be involved.^48^

Supportive data for increased hemolytic activity in pregnancy come from observations of pregnant women with aHUS or paroxysmal nocturnal hemoglobinuria (PNH). Following the introduction of complement inhibiting therapy (eculizumab, an anti‐C5 monoclonal antibody that prevents C5 activation), pregnant women were often found to require increased dosing.^51^

Increased complement activation in pregnancy is likely a normal physiological response consequent on increased placental cellular debris. Enhanced maternal synthetic function may be a normal physiological response to increased consumption, required to maintain effective host protection against infection. Complement homeostasis is key to achieving successful pregnancy milestones, and if this is disrupted adverse events leading to the development of pregnancy‐specific disorders can arise.

## 3 PREGNANCY DISORDERS

### 3.1 Pregnancy‐associated thrombotic microangiopathy

Atypical hemolytic uremic syndrome (aHUS) is a rare kidney disease consequent on AP dysregulation, histologically defined through the presence of a thrombotic microangiopathy (TMA) in the kidney, accompanied with the clinical triad of thrombocytopenia, microangiopathic hemolytic anemia and acute kidney injury.^52^ The majority of aHUS patients carry mutations in proteins of the AP, resulting in its defective regulation. Autoantibodies, primarily those directed against the C‐terminal domains of FH, are also associated with the disease. Many of these autoantibodies block sialic acid‐dependent recruitment of FH to the cell surface, and hence augmentation of CD55‐ and CD46‐downregulation of AP amplification. What is noteworthy in aHUS is that mutations and autoantibodies are predisposing factors for aHUS, and an additional trigger is required for disease to develop.^53^, ^54^, ^55^ Additionally, while aHUS represents an archetypal AP disease, successful therapy with eculizumab (an anti‐C5 monoclonal antibody) identifies C5 activation products (C5a, C5b‐9) as key effector molecules.^56^

Pregnancy‐associated aHUS (p‐aHUS) comes under the umbrella of complement‐mediated aHUS, sharing similar disease severity and frequency of mutations in complement genes.^57^ A large cohort study, incorporating national registries from France, England, and Italy, showed that while p‐aHUS commonly presents in the first pregnancy (58%) and within the postpartum period (76%),^57^ the risk is not significantly lower in subsequent pregnancies. It was hypothesized that cases occurred in the postpartum phase as delivery of the placenta removes the enhanced regulatory capacity of CD55 that the placenta provides. This, coupled with complement activation following parturition, for example through infection and/or bleeding, may provide the trigger that breaches the regulatory capacity of the AP, leading to the clinical manifestation of aHUS. The study found that two thirds of p‐aHUS women required dialysis, with more than 50% reaching end‐stage renal disease (ESRD).^57^ In this context, it should be noted that the data were collated over four decades, with most cases from the pre‐eculizumab era. A more recent study, in the era of eculizumab, showed significant improvement in renal outcomes in women with aHUS triggered in pregnancy.^58^, ^59^ However, even today, affected women will experience an acute aHUS episode before anti‐C5 therapy can be introduced.

In the combined registry study discussed above, an AP‐relevant variant complement protein was detected in 56% of the registry patients, with FH variants being the most abundant; this mirrors the picture seen in complement‐mediated aHUS cohorts.^60^, ^61^ Around 70%–75% of the women had no personal or family history of aHUS and thus could not be identified as being “at risk.” Patients with complement protein variants had more severe outcomes than those in which no variant protein could be identified. The risk of recurrence of aHUS in a subsequent pregnancy is about 25%, and women should be informed of this.^62^

While the hypothesis discussed above, that p‐aHUS is consequent on a decrease in AP regulatory capacity combined with an increased challenge, makes theoretical sense, there is little directly supportive data, and the precise mechanism(s) by which pregnancy triggers aHUS remains unknown. One possibility is that the gravid endothelium, and its altered imbalance of angiogenic factors, may make it more vulnerable to complement activation. Further work investigating the differences between the gravid‐ and non‐gravid endothelium and its ability to control AP activation are needed for rational preventative and treatment strategies to be developed.

### 3.2 Preterm birth

Spontaneous preterm birth (birth between 20^0/7^ and 36^+6/7^) is one of the leading causes of perinatal morbidity and mortality worldwide, with approximately 11% of neonates across the world being born prematurely.^63^ Not only is there an increased rate of neonatal and infant mortality in preterm births, an increase in all‐cause mortality accompanies the child into adolescence.^64^ Surviving infants are at increased risk of impaired development, behavioral problems, and long‐term general health morbidities such as cardiometabolic disease in adulthood.^65^ Given these long‐term health consequences can transcend for decades, preterm birth (PTB) is an important public health issue.

Multiple risk factors that can identify vulnerable women with a predisposition to PTB have been defined or proposed. One possible player is complement, and the studies that implicate a causative role for complement in preterm birth are discussed below.

#### 3.2.1 Murine studies

Inducible mouse models of PTB include vaginal administration of a low‐dose endotoxin (LPS) or of a progesterone antagonist (RU486), both of which lead to cervical ripening and PTB.^66^, ^67^ In both models, increased cervical deposition of C3 was observed; this was accompanied by macrophage infiltration into the cervix. While, in the mouse, the mechanism responsible for cervical C3 deposition is not understood, an increase in serum levels of the anaphylatoxins C3a and C5a, when compared to gestational age‐matched controls, was also noted. An increase in plasma C5a correlated with an increase in cervical matrix‐metalloproteinase 9 (MMP‐9) activity, collagen breakdown, and cervical remodeling. In vitro studies with murine macrophages showed that MMP‐9 release was triggered by C5a.^7^, ^66^ Consistent with this, mice deficient in C5aR did not show an increase in MMP‐9, did not undergo cervical remodeling, nor was premature delivery seen following either LPS or RU486 administration. Progesterone administration also prevented C5a‐induced activation of macrophages and reduced PTB in mice.^66^ Importantly, no local C3 deposition on the cervix, nor evidence of systemic activation of complement in maternal plasma, was identified in mice that delivered at term, indicating that complement activation does not play a role in normal parturition.

#### 3.2.2 Human studies

PTB is associated with intrauterine infection and inflammation. A recent study by Chan et al. has hypothesized a mechanistic role for complement in microbial‐driven inflammation and PTB. The longitudinal study analyzed vaginal microbiota and cervicovaginal fluid, finding, an increase in C3b and C5a in women who delivered preterm. An increase in C3b and C5 were in response to a specific vaginal microbial composition and could be positively correlated with pro‐inflammatory cytokines, providing a link between the microbial environment, complement and PTB.^68^

An early study investigated possible markers of intrauterine infection in amniotic fluid obtained by amniocentesis of women in preterm labor, and found that C3 levels gave the best correlation with infection. This study was performed in women with singleton pregnancies, with amniocentesis performed between 23 and 35 weeks gestation, and found that the culture positive group had higher C3 than the culture negative group.^69^ A number of related studies, all supportive of Elimian et al.,^69^ have subsequently been published. Women with preterm labor associated with intrauterine infection were found to have higher amniotic fluid levels of C3a, C4a and C5a than women with preterm labor in the absence of infection.^70^ The same study found that elevated maternal plasma levels of C5a were also associated with PTB with accompanying intra‐amniotic infection (IAI), whereas maternal plasma levels of C5a in PTB without IAI, and in those who delivered at term, were not elevated.^70^ A follow‐up study by the same group showed that spontaneous labor at term is not associated with changes in amniotic fluid of any of the anaphylatoxins.^71^ Indeed, women with spontaneous labor at term had lower median maternal plasma C5a concentrations when compared to women at term not in labor (no difference was identified in C3a and C4a).^70^ All of this suggests that complement activation fragments could be biomarkers to detect subclinical intrauterine infection. However, their use, is unlikely to change clinical practice due to the practical limitations of obtaining appropriate samples.

Of particular relevance to this review, the activation fragment of FB, Bb, has also been measured in amniotic fluid, with the finding of higher median concentration of Bb in pregnancies complicated with IAI, irrespective of membrane status.^72^ This implies that the host response against IAI infection involves AP activation. Interestingly, a significant relationship was also found between elevated plasma levels of Bb in early pregnancy and spontaneous preterm birth. Plasma levels of Bb in the first trimester of pregnancy were predictive of PTB at less than 34 weeks’ gestation (but not of PTB between 34 and 37 weeks).^73^ One interpretation of this finding is that Bb is generated during an early infection‐related inflammatory event, precipitates changes that lead to PTB, but that the infection remains occult until the onset of preterm labor.

#### 3.2.3 Therapeutic approaches to PTB

The above data suggest a number of possible approaches that could be evaluated for prevention of PTB. The mouse data suggest that both a C5aR antagonist and progesterone might be efficacious.^66^ Until now, no C5aR antagonist has been evaluated, but clinical trials have examined the efficacy of daily vaginal progesterone or weekly intramuscular 17‐alpha hydroxyprogesterone caproate (17‐OHPC) administration to prevent PTB and associated neonatal adverse outcomes, with conflicting results.^74^, ^75^, ^76^, ^77^, ^78^, ^79^ A recent systemic review and meta‐analysis found that vaginal progesterone reduced the risk for PTB before 34 weeks’ gestation and reduced neonatal outcomes in high‐risk singleton pregnancies,^80^ and vaginal progesterone is now recommended by the UK National Institute for Health and Care Excellence (NICE) to prevent early miscarriage.

### 3.3 Pre‐eclampsia

Pre‐eclampsia is a serious complication of pregnancy, occurring in 2%–8% of pregnancies, and manifesting as a pregnancy‐induced onset of hypertension, renal dysfunction, and a myriad of other complications. Untreated, it has serious implications for the health of both mother and fetus.^81^ It has been proposed that pre‐eclampsia develops as a two‐stage process. Stage 1 occurs early in the first trimester, with failed remodeling of the maternal spiral arterioles; this leads to placental Ischemia.^82^ Stage 2 occurs later in pregnancy. In healthy pregnancy, the fetoplacental unit produces the pro‐angiogenic factors vascular endothelial growth factor (VEGF) and placenta growth factor (PIGF). However, once the placental is functionally established, it produces soluble fms‐like tyrosine kinase 1 (sFLT‐1), an anti‐angiogenic factor which sequesters free VEGF and modulates its activity, rendering both VEGF and PIGF inactive.^83^ A delicate balance is required between pro‐ and antiangiogenic factors for maintenance of a healthy placenta.^84^ In pre‐eclampsia, with advancing gestational age (second and third trimesters), this balance is disrupted (Stage 2), and favors the release of sFLT‐1 and other antiangiogenic factors. These spillover into the maternal circulation, leading to multi‐organ maternal endothelial cell dysfunction, organ injury, and the clinical manifestations of pre‐eclampsia.^82^ Interestingly, the kidney again appears particularly susceptible with hypertension and proteinuria being cardinal features.^82^

Over the last two decades, both animal models and human studies have suggested a role of complement in the pathogenesis of pre‐eclampsia. However, what has been difficult to establish is whether complement activation might play a central role in the disease mechanism, or whether it is simply a secondary response to immune dysregulation of placental angiogenesis. Human studies have been confounded by sample size, methodology, disease severity, and gestational age.

#### 3.3.1 Insights into animal models

Given the remit of this review we will focus on studies that implicate the AP in pre‐eclampsia pathogenesis; animal models of the classical (*C1q−/−*) and lectin (*MBL‐A−/−*) pathways will not be discussed.

The BPH/5 (blood pressure high/5) mouse is a spontaneous model of abnormal placental development, and recapitulates much of the clinical phenotype of pre‐eclampsia.^85^, ^86^, ^87^ It has been proposed that complement activation provides an initiation mechanism for the development of pre‐eclampsia as abnormal placentation can be reversed by inhibiting complement activation at the maternal fetal interface.^86^ To investigate this, the effects of two fusion proteins, CR2‐Crry and CR2‐FH, designed to provide targeted complement inhibition at the cell surface, were investigated. The CR2 (complement receptor 2) component targets the fusion proteins to sites of C3 (C3b, iC3b, and C3d) deposition, Crry and FH provide the complement regulatory function. CR2‐Crry inhibits the classical, lectin and alternative pathways, in comparison with CR2‐FH that specifically inhibits AP activation. At the doses used in these studies, the fusion proteins had only minimal effect on systemic complement activity, with preferential targeting to sites with localized complement activation. The constructs remained in situ and functionally active for prolonged periods. Both inhibitors prevented fetal loss and growth restriction in the BPH/5 mice. Increased placental weight and normalized placental spiral artery morphology were achieved with CR2‐Crry treatment, but not in mice treated with CR2‐FH,^86^ suggesting that, while the AP plays a role in this early stage of placentation, activation of other pathways is also important. Increased placental VEGF concentrations were achieved with both agents. Complement activation at the maternal‐fetal interface leads to neutrophil recruitment, elevated local tumor necrosis factor‐alpha (TNF‐α), reduced VEGF, abnormal placentation, and fetal demise. The above data suggest that complement activation, and the ensuing neutrophil infiltration into the placenta, triggers abnormal spiral artery remodeling and angiogenic dysregulation.^86^

In an inducible rat model of placental insufficiency (reduced uterine placental perfusion through surgical manipulation), hypertensive rats had decreased circulating C3 and increased circulating C3a, showing that complement, specifically C3, activation had occurred.^88^ Soluble complement receptor 1 (sCR1) is a synthetic form of CR1 lacking its transmembrane domain, and an inhibitor of the CP, LP, and AP C3 and C5 convertases. Administration of sCR1 in this rat model inhibited complement activation, with reduced circulating C3a and a reduction in mean arterial pressure (MAP).^88^ While VEGF was reduced in hypertensive rats, sCR1 inhibition of complement activation had no effect on this.^88^ In further evaluation of potential mediators of pre‐eclampsia‐like effects, administration of both C3aR and C5aR antagonists was shown to inhibit the increase in MAP, with both antagonists being equivocal. Surprisingly, combination therapy failed to augment their effects. However, only the C5aR antagonist was able to attenuate endothelial dysfunction, suggesting that C3a and C5a have specific immunological roles in contributing to placental ischemia.^89^ In agreement with sCR1 data, neither antagonist affected VEGF levels.^89^ Thus, complement activation leading to placental ischemia‐induced hypertension was independent of VEGF in this inducible model.

The difficulties of interpreting animal models of pre‐eclampsia are illustrated in a further mouse model. Mating of two mouse strains, CBA/J × DBA/2, reproduces features of pre‐eclampsia with proteinuria, renal impairment (elevated BUN), glomerular fibrin deposition and glomerular endotheliosis.^90^ A single dose of CR2‐Crry at 5 dpc prevented development of proteinuria and glomerular endotheliosis.^90^ However, in contrast to the inducible rat model, an increase in C5a levels was associated with disruption to the balance of angiogenic factors, with elevated C5a correlating with reduced VEGF and increased sFLT‐1. This argues for a role for C5a in the induction of anti‐angiogenic factor release from the inflamed and ischemic placenta.^91^

In a different approach, the effect of injection of IgG from normotensive or pre‐eclamptic pregnant women into pregnant C57BL/6 mice was examined. IgG was purified from the blood of pre‐eclamptic women with severe disease (gestational age 32 weeks) and from women with uncomplicated normotensive pregnancies (gestational age 36 weeks), and injected into pregnant mice at 13 and 14 dpc, followed by euthanasia at 18 dpc. Histological features of placental distress were identified in the mice injected with IgG from pre‐eclamptic women, but not in mice injected with IgG from normotensive women. Pre‐eclamptic women have agonistic autoantibodies to the angiotensin II type 1 (AT1) receptor, and in this study, it was demonstrated that C3 deposition in placentas of mice injected with IgG from pre‐eclamptic women correlated with AT1 receptor activation.^92^ While the mechanism by which AT1 receptor activation induces C3 activation is not known, it seems clear that C3 activation plays an important role in initiating events that lead to pre‐eclampsia. Blockade of the C3aR with a C3aR antagonist, SB290157, reduced hypertension and proteinuria in the mice, prevented intrauterine growth reduction (IUGR), and reduced sFLT1, demonstrating a role for C3aR signaling in impaired placental angiogenesis.^92^

#### 3.3.2 Complement activation within the placenta

Early studies identified increased C3 and C9 deposition in pre‐eclamptic placentas when compared to normotensive controls.^44^, ^93^ However, a more recent study comparing early onset (<34 weeks of gestation; 7 subjects) vs. late onset pre‐eclampsia (5 subjects) vs. normotensive controls (10 subjects) failed to find supportive data for this; activated C4 deposition, an indicator of CP and/or LP activation, was abundant, but no difference in C3b/IC3b/C3d or C9 deposition was seen between the groups.^94^ In the same study, abundant amounts of the membrane‐bound regulators CD55, CD46, and CD59 were found, both in pre‐eclamptic placenta and healthy controls. There was, however, an irregular distribution of C4bp and FH in pre‐eclamptic placenta,^94^ suggesting possible structure‐specific regulatory control needs.

In a separate study, a significant increase in mRNA expression of CD55 and CD59 in pre‐eclamptic placenta was observed.^95^ This, coupled with diffuse placental C4d deposition associated with a significantly lower gestational age at delivery, is suggestive of excessive CP activation and an altered need for a compensatory increase in membrane protection in the pre‐eclamptic placenta. Interestingly, in this study, no difference in staining for properdin between pre‐eclamptic and normotensive placentas was seen, possibly indicative of a minimal role for the AP in complement‐mediated events.

A reduction in C3aR mRNA and protein expression in placental tissue of severe early onset pre‐eclamptic women compared with preterm normotensive control pregnancies has also been reported.^96^ This reduction in C3aR expression suggests impairment in C3a‐mediated effector mechanisms, seemingly at odds with the data reported by Wang et al.,^92^ who concluded that C3a drove pre‐eclampsia. Variable results have also been obtained with analysis of C5aR expression, with no change,^96^ elevated,^97^ or reduced^98^ expression in pre‐eclamptic placentas. In vitro work has also shown C5a is able to disrupt the angiogenic balance within the placenta, with C5a stimulated trophoblasts increasing expression of sflt‐1 and decreasing PIGF.^97^

No clear picture, other than that complement activation, and its probable aberrant regulation, plays a role in the development of pre‐eclampsia, emerges from the above data. Certainly, there is no clear evidence pointing to a role of the AP. It should, however, be noted that the above studies were of small cohorts, and made at different pre‐eclamptic stages, and the large spectrum of disease seen in pre‐eclampsia may account for the conflicting data.

#### 3.3.3 Complement activation in the maternal circulation

A pivotal prospective study by Lynch et al., of 701 pregnant women, identified Bb as a biomarker for women at increased risk of pre‐eclampsia. Women with elevated plasma levels of Bb before the 20th week of pregnancy were three times more likely to develop pre‐eclampsia than those with normal levels of Bb at the same time point.^99^ In a follow‐up study, plasma Bb levels were measured longitudinally during pregnancy (up to three samples/pregnancy). Of the 552 evaluable pregnancies, 28 (5%) developed pre‐eclampsia; these were compared with randomly selected normotensive pregnancies from the same population. The study found that Bb levels were highest, in both normotensive and pre‐eclampsia subjects, in early pregnancy, with a gradual decline up to the time of parturition. While the initial plasma level of Bb varied with gestational age at blood draw, a significant trend to increased Bb levels between 10 and 21 weeks was found in women who subsequently developed pre‐eclampsia when compared to women who remained normotensive during pregnancy. No relationship between Bb levels in later pregnancy and pre‐eclampsia was identified.^100^

This was confirmed by Derzsy et al., who looked at complement protein levels at a late gestational stage, and found that pre‐eclamptic (*n* = 60; gestational age at blood draw 36 weeks) pregnancies had higher CRP, C4d, C3a, and sC5b9, and lower C3, when compared to healthy pregnant controls (*n* = 60; gestational age at blood draw 37 weeks). When both pregnancy groups were compared with healthy non‐pregnant controls, FH levels were also found to be higher, and C1inh levels lower, in pregnancy, with or without pre‐eclampsia. No difference in plasma Bb (or C4bp) levels between the pregnancy groups, or between these and healthy non‐pregnant women, was observed at this late stage in pregnancy.^49^ In further analysis of complement protein plasma levels in these groups, Derzsy et al. also noted that pre‐eclamptic pregnancies with intrauterine growth restriction (IUGR) had higher sC5b‐9 than those without IUGR. Taken together, these data suggest that in late pregnancy there is elevated activation of complement, probably through both the CP and the LP, but that only C5b‐9 generation is exacerbated in some cases of pre‐eclampsia.

Ma et al. found an increase in maternal C5a in women with pre‐eclampsia, and this positively correlated with maternal blood pressure and arterial stiffness.^97^ These results were echoed in further studies showing an increase in maternal circulating C5a in pre‐eclamptic women compared with normotensive controls.^101^, ^102^, ^103^ Soto et al. measured C3a, C4a, and C5a plasma levels in normotensive and pre‐eclampsia pregnancies and found that pre‐eclampsia was associated with increased plasma concentration of C5a (but not C3a or C4a). They also compared complement anaphylatoxin levels in small for gestational age (SGA) pregnancies, with or without pre‐eclampsia, with the surprising finding that whereas SGA associated with pre‐eclampsia was associated with elevated C5a levels, SGA alone was not.^104^ Pre‐eclampsia associated with IUGR indicates placental insufficiency, thus terminal complement activation may be consequent on debris generated by the failing placenta. Systemic complement activation in late pregnancy may therefore simply reflect a pro‐inflammatory environment in the placenta and the sequelae of maternal endothelial dysfunction, that is, an association with, rather than causation of, IUGR. A lack of consistent association between plasma C3a levels and pre‐eclampsia has also been noted by others.^102^

While pre‐eclampsia is almost invariably associated with elevated maternal plasma C5a, the data with sC5b‐9 are less consistent. As noted above, Derzsy et al. found elevated sC5b‐9 levels in late stage pregnancies with pre‐eclampsia, and especially with pre‐eclampsia associated with IUGR.^49^ By contrast, Haeger et al., in a longitudinal study of 685 primiparous women followed throughout pregnancy, only found an increase in sC5b‐9 in women with HELLP syndrome at delivery.^103^ (HELLP ‐Hemolysis, Elevated Liver enzymes, Low Platelet syndrome is a serious blood clotting complication of pregnancy, which, while usually occurring at delivery, can occur earlier in pregnancy.) Lynch et al. conducted a secondary analysis to see if sC5b‐9 could be used as an independent predictor of pre‐eclampsia at 10–15 weeks’ gestation, but no difference was identified in early pregnancy among women who later develop pre‐eclampsia when compared to normotensive pregnant controls.^105^ Burwick et al. looked at sC5b‐9 levels in women with severe pre‐eclampsia, comparing these with controls with pre‐existing chronic hypertension and to healthy controls, both matched by parity and by gestation age (±2 weeks). They found an increase in urinary excretion of sC5b‐9 which correlated with elevated sflt‐1 (anti‐angiogenic) and suppression of PIGF and VEGF (pro‐angiogenic) factors in plasma,^106^ but no association between plasma sC5b‐9 and the same angiogenic factors. Additionally, while plasma levels of sC5b‐9 were increased in cases of severe pre‐eclampsia when compared to healthy controls, there was no difference between the pre‐eclampsia and chronic hypertension group.^107^

While it is difficult to extrapolate plasma protein levels with local activation events, elevated plasma C5a is consistently seen in pre‐eclampsia. This association does not define C5 activation as causative of pre‐eclampsia, but a causative association would be consistent with in vitro work that has shown that C5a induces monocytes to produce sFLT‐1 (soluble VEGF receptor 1) which then sequesters VEGF. This was confirmed with in vivo data showing that the complement system regulates VEGF activity, and that a deficiency of free VEGF induced by high levels of sflt‐1 leads to abnormal placental development.^91^

A surprising, but potentially important, finding arising from evaluation of complement activation products as markers for pre‐eclampsia is that urine, rather than plasma, might provide a better biomarker source material.^106^

What also appears clear from evaluation of complement activation products in pre‐eclampsia is that it is early (first trimester) complement activation, and particularly FB activation, that is predictive of pre‐eclampsia later in pregnancy. It also points intriguingly to the possible involvement of the AP in the defective placentation that leads subsequently to pre‐eclampsia.

#### 3.3.4 Genetic studies

It has been hypothesized that polymorphisms in complement genes may be linked to adverse pregnancy outcomes. The PROMISSE study looked at 250 pregnant women with a diagnosis of SLE and/or APS; this population have an increased risk of developing pre‐eclampsia, and it was hypothesized they might have an impaired ability to regulate complement activation. In this study, of 250 pregnancies, *CD46*, *CFI*, and *CFH* genes were sequenced.^108^ These proteins are regulators of C3 activation. Of these 250 pregnancies, 40 developed pre‐eclampsia, and, of these, seven (17%) were found to be heterozygous for a variant previously identified as a risk variant for aHUS. Five risk variants of CD46 or FI and one novel CD46 variant were identified. While these proteins are involved in regulation of both CP and AP C3 convertases, the fact that aHUS is clearly a disease of dysregulated AP activation^109^, ^110^, ^111^ enabled the authors to conclude that the AP amplification loop plays a role in susceptibility to pre‐eclampsia.^108^ That said, no FH risk variants were identified, and of the three regulators investigated, only FH is AP‐restricted. What is also notable in this study is that in 83% of the pre‐eclamptic women, no AP risk variant was identified. It is possible that some carried risk variants in genes not evaluated, but, at first sight, it would seem that only a minority of women who went on to develop pre‐eclampsia had a clearly definable complement‐dependent risk, suggesting a high degree of complexity in determinants of the condition. It should also be noted that this study was conducted in women with an underlying autoimmune condition that predisposes them to complement activation.

In contrast to the above findings, Lokki et al. sequenced the CD46 exome in 95 Finnish women, and found no association between CD46 risk variants and severe pre‐eclampsia.^112^ The two studies are not equivalent in view of the autoimmune status of the PROMISSE population and ethnic differences between the two groups, but this divergence in findings demonstrates the need for further work to replicate (and extend) evaluation in a group without underlying autoimmune disease, and who will likely have a higher threshold for developing pre‐eclampsia.

A recent study has suggested the possibility of a pregnancy‐specific complotype predisposing women to development of pre‐eclampsia. In this study, *CFH*, *C3*, and *CD46* genes were sequenced. A novel approach in this study was to focus on fetal, not maternal, *CD46*, and hence introduce consideration of the allogeneic nature of the fetal‐placental interface. The allelic frequencies of two SNPs in fetal *CD46* were elevated in pre‐eclampsia pregnancies when compared with normotensive pregnancies. These, combined with allelic variants of *C3* and *CFH*, allowed complotypes defining susceptibility to pre‐eclampsia to be defined. What was also noteworthy in this study was that the rare CD46 risk alleles are associated with decreased CD46 expression. Consistent with defective regulation of C3 activation, C4, C3, and C5b‐9 deposition on the placenta was significantly higher in pre‐eclamptic vs. normotensive pregnancies.^113^

#### 3.3.5 Long‐term sequalae of pre‐eclampsia

Pre‐eclampsia is primarily a disease of the endothelium, and women who suffer from pre‐eclampsia have a greater risk of cardiovascular disease in later life. Large population‐based studies have shown an increased risk of ischemic heart disease, hypertension, thromboembolic events, and type 2 diabetes (T2DM). The severity of pre‐eclampsia dictates the severity of cardiovascular disease developing in later life.^114^ Cardiovascular disease is the leading cause of death in women, so there is a vital need to understand how pre‐eclampsia leads to persisting endothelial damage. Is it causally associated with postpartum vascular remodeling,^115^ or does it unmask women with a pre‐disposition who have failed the “stress test” of pregnancy? There is a paucity of data investigating the role of complement on long‐term vascular remodeling following pre‐eclampsia. It has been shown that hypoxia‐induced perivascular inflammation is complement‐dependent, and that AP activation plays a critical role in this.^116^ Recent in vitro work has demonstrated higher AP activation and C3a generation by human glomerular vascular endothelial cells (GMVECs) compared with brain microvascular endothelial cells (BMVECs),^117^ showing that not all endothelial cells are equivalent. One avenue that deserves further evaluation in pre‐eclampsia is the role that the AP has in postpartum vascular remodeling^118^ in different tissues.

#### 3.3.6 Treatments for pre‐eclampsia

As outlined in this section, there is a plethora of evidence pointing to dysregulation of complement activation, and particularly of C5 activation, in the inflammatory‐mediated injury of pre‐eclampsia. Eculizumab, a monoclonal antibody inhibitor of C5 activation, has been highly successful in the treatment of PNH and aHUS. While both are AP‐driven diseases, the major effector arm of both conditions requires C5 activation. The latter similarity with pre‐eclampsia suggests that pre‐eclampsia might be effectively treated by anti‐C5 therapy. The very narrow species‐specificity of eculizumab precludes preclinical evaluation in a pharmacodynamically relevant species, and hence, no preclinical reproductive and developmental safety evaluation has been possible.^119^ However, continuation of treatment during pregnancy of patients with PNH and aHUS has shown an acceptable safety profile for the drug during pregnancy.^62^, ^120^ Additionally, despite eculizumab binding to the Fc receptor, and being found in one third of cord blood samples, it does not accumulate in fetal plasma. The newborn's complement activity remains within the normal range despite C5 activation in the mother being adequately blocked,^121^ even though increased dosage might be required during pregnancy because of increased C5 synthesis and a higher distribution volume.^62^ Importantly, eculizumab does not appear to transfer to the newborn via breastmilk.^120^ A number of reports of the successful treatment of pre‐eclampsia with eculizumab have been published.^122^, ^123^ However, there are also an increasing number of reports of pregnancy hypertension syndromes (including pre‐eclampsia) in aHUS despite adequate complement blockade with an anti‐C5 therapy, so it is unlikely that eculizumab therapy will be a panacea for pre‐eclampsia.

Aspirin is the therapy proven to reduce the incidence of preterm pre‐eclampsia, and there is a rationale for this.^124^ A recent study examining hepatocyte nuclear transcription factors (HNF), found that HNF4α positively regulated expression of C3 and FB. Both were significantly downregulated in transgenic placentas by aspirin treatment in a mouse model of severe pre‐eclampsia induced by feto‐placental overexpression of human transcription factor STOX1A.^125^

Heparin has therapeutic effects beyond anticoagulation. Unfractionated or low‐molecular‐weight heparin prevented complement activation and adverse obstetric complication in a mouse model of APS. This effect was distinct from its anticoagulation properties as alternative anticoagulants did not replicate the protective effects.^126^ Clinical trials with heparin have also shown inconsistent results in pre‐eclampsia. Collating evidence through systematic reviews and meta‐analyses has failed to provide a consensus; it is likely that the heterogeneity of the condition explains the conflicting data. Women who present with severe early onset disease, identified within the index pregnancy using a combination of placental and cardiovascular assessment, may identify a cohort whom would benefit from the protective vascular effects of heparin, but further clinical studies are required to assess this.^127^

It is also worth noting that none of the above therapies are preventative of disease, but treat disease once it occurs. Any early preventative treatment would have to be introduced early in pregnancy, and for this, a highly reliable diagnostic of early disruption to placentation would be required.

### 3.4 Obesity and adverse effects of pregnancy

Pregnancy is associated with a physiological state of inflammation and insulin resistance, which is further amplified in women who are overweight.^128^ Obesity is a known risk factor for pre‐eclampsia, with a higher incidence of pre‐eclampsia in women who are obese.^128^ Weight loss preceding pregnancy reduces the risk of pre‐eclampsia.^129^ Obesity also increases neonatal adverse events including macrosomia (large for gestational age) and congenital defects.^130^ Obesity is the expansion of adipose tissue, and complement components, particularly C3, FB and Factor D (FD),^131^, ^132^ can be synthesized by adipose tissue. Of particular relevance here, the primary site of FD synthesis is adipose tissue, and its role in adipose tissue metabolism (as adipsin) has long been recognized.^133^ An increase in local concentrations of C3, FB, and FD is known to promote local complement activation.^134^ Possibly even more relevant, elevated FD levels result in increased AP activation.^135^ Complement activity is increased with obesity, positively correlating with BMI and decreasing with weight loss.^84^ In mice, an increase in C3 is found in the adipose tissue before and during pregnancy as well as at the maternal fetal interface; this is reduced following calorie restriction, implying that dietary intervention could influence the propensity of immune dysregulation during pregnancy.^84^, ^136^ C3aR is highly expressed in adipose tissue and, in mice, with increased expression when they are placed onto a high fat diet.^137^ In mice, maternal obesity has been shown to directly alter the placental transcriptome during development.^138^ Women with increased concentrations of Bb and C3a combined with obesity were more likely to develop pre‐eclampsia.^105^, ^128^

In addition to its value in investigating the mechanism of pre‐eclampsia, the BPH/5 mouse model is useful in study of the effects of obesity on complement activation. The female BPH/5 is obese with borderline hypertension but an absence of renal disease. Pregnancy exacerbates the model, female mice developing a superimposed pre‐eclampsia.^136^ Adult females also show hyperphagia and hyperleptinaemia with signs of leptin (an appetite‐suppressing hormone) resistance. Interestingly, males are not obese and do not mirror the cardiometabolic phenotype of their female counterparts.^136^ Following blastocyte implantation, and prior to decidualization and placentation, BPH/5 mice have reduced macrophage but increased T lymphocyte infiltration within the maternal decidua, and reduced IL‐10 levels, indicating an aberrant maternal immune response.^139^ At 7.5 dpc complement gene upregulation (*C3* and *CFB*) in BPH/5 mice corresponds to peak decidualization.^85^ An increase in placental C3 mRNA expression is detected with increased deposition of C3 and C9 at 10.5 dpc localized to the trophoblast giant cell layer. Increased mRNA expression of VEGF‐α at 5.5 dpc preceded complement activation; this correlated with abnormal placental vasculaturisation, and culminated in downregulation of pro‐angiogenic genes and an increase in levels of the anti‐angiogenic factor, sFLT‐1, at 10 dpc.^85^ Thus, early local altered expression of VEGF‐related genes and abnormal decidual vasculaturisation preceded Sflt‐1 upregulation and increased complement deposition. Abnormal decidual angiogenesis, with associated complement activation, appears to contribute to altered trophoblast invasion and subsequent poor placentation of the BPH/5 mouse.^85^

Visceral white adipose tissue (WAT) contains inflammatory mediators, including complement factors, that, particularly when adjacent to the female reproductive tract, may contribute to angiogenic imbalance and pre‐eclampsia.^84^ Using the BPH/5 mouse model, investigators sought to determine whether reversal of maternal obesity through calorie restriction lowered inflammation in adipose tissue, and if this improved placental development. C3 and FB (but not FD) mRNA expression is increased in the reproductive WAT of non‐pregnant BPH/5 females when compared with a matched cohort of calorie‐restricted mice, showing that the reproductive WAT may be a source of complement components. As calorie restriction was successful in reducing mRNA expression of C3 and FB in the reproductive WAT of non‐pregnant females, it was thought that reducing obesity could attenuate the inflammatory milieu in the reproductive WAT. BPH/5 mice were therefore calorie‐restricted for 2 weeks prior to, and for the first seven days of, pregnancy. This attenuated C3 gene expression, but not that of FB or FD expression, at the implantation site.^140^

Calorie restriction during pregnancy also restored VEGF and PIGF mRNA levels at the implantation site in the BPH/5 mouse.^140^ While interpretation of the above data is complex, it appears that maternal reproductive WAT is a source of increased C3 during pregnancy, especially at the maternal‐fetal interface, and that reducing maternal weight, and hence C3 (from which antiangiogenic factors are produced) in adipose tissue, may assist in improving the angiogenic balance in the BPH/5 mice.

Correa et al. compared immunological and metabolic blood components during the first half of pregnancy in women who began a pregnancy under normal healthy conditions with those who had risk factors for obstetric complications, including obesity.^141^ Pregnancy outcomes, ranging from pre‐eclampsia to SGA deliveries, were significantly higher in pregnancies initiated in women with risk factors. While no changes in AP‐specific proteins (Bb, FD) were observed, elevated CRP, C4, and C3a were seen in the at risk group, implying CP and/or LP activation.^141^ It has been suggested that higher CRP levels may have an impact on the regulatory activity of FH, impairing its ability to clear apoptotic cells.^101^ CRP binds to FH SCRs 7 and 8–11 in a calcium dependent manner.^142^ These sites are distinct from C3b binding sites; CRP preserves the ability of FH to enable FI‐mediated cleavage of C3b, and it has therefore been proposed that it might target functionally active FH to injured self‐tissue, restricting excess complement attack in tissues without affecting AP activity against invading microbes in the blood.^142^ In the context of pregnancy in obese and other at risk populations, it is possible that CRP‐dependent sequestering of FH might constitute a maternal response that offers some protection against complement attack on the placenta.

### 3.5 Gestational diabetes

Gestational diabetes (GDM), in which pregnant women with no known history of diabetes develop chronic hyperglycemia, is one of the most common complications of pregnancy. GDM can lead to serious maternal and fetal complications. While it typically resolves upon delivery, it is associated with T2DM and cardiovascular disease in later life. It also increases the risk of future obesity, cardiovascular disease, T2DM and GDMs in the infant (reviewed in Ref. 143).

In normal pregnancy, a state of insulin resistance develops to facilitate the delivery of glucose to the placenta, supporting the growing fetus. GDM arises during pregnancy due to impaired β cell function on a background of chronic insulin resistance; low‐grade inflammation has been involved in the pathogenesis of insulin resistance. In T2DM, activation of the AP may participate in development of a chronic inflammation and insulin resistance state. *CFB* gene expression is higher in the omentum tissue, with a positive correlation with BMI and fasting glucose,^144^ and higher circulating levels of FB (and properdin) have been identified in T2DM patients and their first‐degree relatives compared with a healthy control group.^145^, ^146^ These data point directly to a role of FB, and hence the AP, in the development of GDM. As GDM is considered a precursor for T2DM, it is plausible to hypothesize a role of the AP in the pathogenesis of T2DM as well.

Work in a rat model of spontaneous hypertension (exhibits hypertension, insulin resistance and dyslipidemia, features of metabolic syndrome) showed that knocking out the *CFB* gene improved glucose tolerance and insulin sensitivity, with redistribution of visceral to subcutaneous fat. This suggests an important role, at least in the rodent, for FB in development of metabolic syndrome,^147^ yet another risk factor for diabetes. Additionally, a reduced blood pressure, increased ejection fraction, and reduced left ventricular mass was seen in the *CFB* knockout, implying a wider role in cardiovascular disease.

While studies linking GDM to complement activation are limited in number, one has shown that higher levels of circulating Bb between 11 and 17 weeks of gestation are associated with an increased risk of developing GDM.^148^ FH levels are also increased in patients with T2DM and plasma levels of FH are negatively associated with insulin sensitivity,^144^ suggesting an increased regulatory requirement in these conditions. In a follow‐up nested case control study of 397 pregnant women, the same group analyzed FH levels in women who had GDM, comparing them with non‐GDM controls, finding FH levels to be higher in GDM. FH levels were not, however, independently associated with GDM.^149^

Screening and identifying women with GDM enables early treatment to reduce maternal and fetal morbidity. A secondary analysis from the pregnancy exercise and nutrition research study (PEARS) found that the maternal C3 levels were predictive of not just GDM, but also pregnancy‐induced hypertension and pre‐eclampsia.^150^ Associations were, however, weak, and it is difficult to draw any firm conclusions from them.

Low levels of CD59, the membrane‐bound inhibitor of the complement membrane attack complex, are shed into blood. Hyperglycemia results in a non‐enzymatic glycation of CD59, which inactivates the inhibitor. The plasma level of glycated CD59 has been investigated as a potential early diagnostic biomarker of GDM (at <20 weeks’ gestation), with promising results. Soluble glycated CD59 has also been associated with LGA infants.^151^ Glycation and inactivation of placental CD59 may result in increased complement‐mediated placental damage by the membrane attack complex, potentially contributing to the multiple complications seen in women with GDM.^152^

## 4 ROLE OF ALTERNATIVE PATHWAY AND THE INCREASED OBSTETRIC BURDEN IN WOMEN WITH UNDERLYING SYSTEMIC AUTOIMMUNE DISEASES

In the previous sections, we have discussed the potential role of complement in the development of pregnancy‐specific disorders. We will conclude with discussion of the role of complement, and specifically of the AP, in women with underlying conditions who are at increased risk of complement‐related disorders because of an autoimmune disease, focusing on SLE and APS. These conditions disproportionately affect women of childbearing age, placing them at increased risk of obstetric complication during their pregnancies.

### 4.1 SLE

SLE is a complex multisystem autoimmune disease. Complement activation by immune complexes formed in the fluid phase and at the tissue level following interaction of autoantibodies with their target antigen leads to the clinical manifestations.^153^ Complement activation in SLE leads to a reduction in circulating C3, C4, and C1q, reflecting consumption, with a concomitant increase in split products that correlates with disease activity. Pregnancies in patients with SLE are regarded as high risk due to an increase risk of disease flaring coupled with high risk of adverse pregnancy outcome. Pregnant women with SLE have an increased risk of pregnancy loss, PTB, IUGR, and premature rupture of membranes, with an increase in pre‐eclampsia if their disease is active (13–35% compared with 5%–8% in the general population).^154^ Despite this, improvements in maternal mortality and reduction in fetal mortality have been achieved over the last two decades.^155^ Risk factors for poor pregnancy outcome include hypertension, secondary antiphospholipid syndrome and lupus nephritis. A large systematic review found that one‐quarter of pregnancies were unsuccessful, with 39.4% preterm births within the live birth cohort. Active lupus nephritis increases the risk of adverse pregnancy outcomes^156^; a history of lupus nephritis is associated with a threefold increased risk of renal flare during pregnancy.

In an early paper, Buyon et al. showed that the AP was activated in disease flares in pregnant women with SLE, and that this was associated with a lowered CH50.^157^ What was especially interesting in this study was that elevated Ba levels were also seen in pre‐eclampsia, indicative of AP involvement, but that these were not associated with a low CH50, indicating differences in the triggers for complement activation. Consistent with this, and much later, analysis of complement activation products in the PROMISSE cohort showed that Bb and sC5b‐9 detected in the circulation in early gestation identified women whom later developed an adverse outcome.^158^

Analysis of placentas from antiphospholipid antibody (aPL)‐negative SLE patients has found evidence of increased complement deposition of C4d and C5b‐9 in syncytiotrophoblasts and extravillous trophoblasts when compared to healthy control placentas.^159^ Lupus anticoagulant was associated with adverse pregnancy outcomes after the first trimester, but no association was seen with anticardiolipin antibodies.^160^, ^161^, ^162^

### 4.2 APS

APS is characterized by thrombosis and recurrent pregnancy loss accompanied with detectable antiphospholipid antibodies (aPL). It is the leading cause of recurrent pregnancy loss. In women with a history of obstetric APS, the current recommended treatment is with low‐dose aspirin and low‐molecular‐weight heparin.^163^ In a large European Registry study, the outcomes of pregnancies in 1000 women were analyzed. Miscarriage was recorded in 38.6% of the pregnancies, pre‐eclampsia in 18.1%, and IUGR in 16.1%. However, in those women who received the recommended treatment, a live birth rate of 85% was recorded. This fell to 72.4% in women receiving either aspirin or low molecular weight heparin, but not on the recommended schedule, and to 49.6% in women receiving no treatment. 15.2% of patients on recommended treatment remained refractory.^164^

It should be noted that, since the initiation of the European Registry study, hydroxychloroquine (HCQ), a disease‐modifying antirheumatic drug (DMARD) also used as an antimalarial, has been introduced in the treatment of pregnant women with APS, with some success, and this may impact on the outcomes reported above. HCQ appears to have been introduced based on anecdotal data, with little understanding of its mechanism of action. In investigating this, Bertolaccini et al. demonstrated that it prevented complement activation in vivo and in vitro, with lower C5a levels and reduced placental insufficiency with HCQ‐treatment in a mouse model of APS.^165^ Similarly, HCQ lowered C5a levels in pregnant women with APS. HCQ appears safe to both mother and fetus, and these mouse studies suggest that a possible mechanism of action is blockade of complement (C5) activation, consistent with data reported in earlier sections suggesting that C5 activation early in pregnancy might adversely impact placentation.

Antiphospholipid (aPL) antibodies activate a plethora of immune cells, monocytes, neutrophils, platelets, and endothelial cells; this culminates in inflammation and thrombosis. Branch et al. showed that passive infusion of IgG from patients with aPL increased the rate of fetal resorption in pregnant mice, illustrating the pathogenic role of the antibody in fetal loss.^166^ Holers et al. showed that C3 activation was required for aPL‐induced fetal loss and growth restriction. Pregnant mice were injected with human IgG containing aPL antibodies at 8 and 12 dpc. The animals were then euthanized on day 15 and found to have an increased incidence of fetal resorption (40%) and reduced fetal weights compared with control mice.^167^ Mice treated with a C3 convertase inhibitor (Crry‐Ig) or mice deficient in C3 did not experience fetal loss or fetal growth retardation,^167^ confirming that complement activation is the major mechanism of aPL mediated tissue injury. Follow on work from Girardi et al. using the same model showed that while C4 deficient mice were protected from fetal injury, FB was required for fetal death, confirming a critical role for the AP in the induction of fetal loss.^168^ Confirmation of this came from the use of a novel anti‐murine FB monoclonal antibody that inhibited AP activation. Mice treated with the antibody had significant protection from AP activation, and reduced fetal resorption, decidual inflammation, and decidual complement activation.^169^

Further analysis of the effector mechanism showed that the C5a‐C5aR axis and C5a‐dependent recruitment of neutrophils were key mediators of fetal injury. Both C5aR deficiency and C5aR antagonist administration were protective.^168^ Key sequelae to C5a production are that neutrophils are attracted into the deciduas, endothelial cells are stimulated to produce monocyte chemoattractant protein‐1, and monocytes express tissue factor. The culmination of these (and other related events) is trophoblastic injury and fetal loss. The pathogenicity of C5a, and its potent ability to induce pro‐thrombotic conditions within the deciduas, was confirmed when it was shown that C6 deficient mice had a similar rate of pregnancy loss as wildtype mice,^170^ and therefore that the consequences of C5b‐9 generation are insignificant in this model.

Collating studies using murine models of APS shows that aPL‐induced tissue damage is mediated through complement activation. While amplification via the AP is critical, induction appears to be through the CP, culminating with C5a as the dominant driver of disease pathology. Heparin has been shown to attenuate inflammatory responses through multiple interactions with complement proteins including inhibition of C1q binding to immune complexes, inhibition of C4 activation, and blockade of the C3 convertase in the amplification loop and the formation of MAC.^126^ Heparin, at sub‐anticoagulant doses, prevented obstetric complications by blocking CP activation at the decidual tissue.^126^ Combination therapy with heparin and a FB inhibitor and/or a C5aR antagonist might therefore provide a therapeutic strategy to explore in the treatment of refractory obstetric APS (but see cautionary note below).

The above conclusions regarding the role of complement are all derived from animal model studies, and it is important that corroborative data in human pregnancies be evaluated. An increase in deposition of C4d and C3b on human APS‐placentas when compared with healthy pregnancy controls has been found.^171^ This was apparent even if the disease was clinically senescent with no adverse features. Surprisingly, there was a decrease in C5b‐9 deposition; the reason for this is not understood.^171^ In a separate study, complement activation products were identified in all placentae examined, with C1q, C4 and C3 detected on the decidual endothelium vessels at sites of IgG and IgM deposition, whereas C5b‐9 showed a predominance for a subendothelial distribution.^172^ Complement deposition was also found on surface of syncytiotrophoblasts with additional distribution of C5b‐9 on intervillous fibrin deposits, and C3 deposition on the endothelium of villous vessels.^172^ Interestingly, this occurred despite treatment with low‐dose aspirin and heparin. One conclusion from this study was that the anticomplement effects of heparin seen in the murine model are not replicated in humans.^172^

In the above study, complement deposition was found in all APS placenta, even in women who had a live birth. Antiphospholipid antibodies bind to endothelial cells, inducing activation through upregulation of ICAM, VCAM, E‐selectin, and tissue factor. This, coupled with platelet activation by C3a, C5a and C5b‐9, results in a pro‐thrombotic environment.^173^ It has been suggested that these events overwhelm the complement regulatory capacity of placental CD55, CD46, and CD59, leading to Ischemia and tissue injury.^173^

When comparing circulating complement levels during pregnancy, some multicenter case–controlled studies in pregnant women with APS have shown that lower C3 and C4 at baseline and at the end of pregnancy was significantly correlated with poor pregnancy outcome.^174^, ^175^, ^176^ This, however, has not been a universal finding; while one group identified lower levels of C3 and C4 in each trimester in APS pregnancies when compared with healthy women, this was not associated with adverse pregnancy outcome.^177^ Despite these inconsistencies, hypocomplementaemia is still considered a prognostic factor for adverse pregnancy outcomes in APS patients.^178^

A significant increase in levels and frequencies of FH autoantibodies has also been reported in patients with APS when compared to matched controls, higher in secondary APS than in primary APS. (Secondary APS is APS that occurs on top of a pre‐existing condition, most commonly SLE.) This study also reported that the presence of FH autoantibodies was associated with venous thrombosis.^179^ Given our current understanding of the role that surface‐recruitment of FH plays in regulation of AP activation on host cells (discussed earlier), autoantibodies to the C‐terminal region of FH that block this recruitment might be expected to exacerbate any APL‐antibody effect, further disturbing the orchestrated interplay between complement and the clotting cascade. The evidence linking anti‐FH antibodies to aHUS is compelling, with thrombosis in both aHUS and APS resulting from activation of the endothelium, which generates a pro‐thrombotic phenotype. Supportive of this is the observation that FH levels are lower in APS than in healthy controls.^180^ As yet, no systematic evaluation of the role of autoantibodies to FH in obstetric APS has been published, but one would be warranted, if only to identify women at increased risk of obstetric complications.

In addition to adverse pregnancy outcomes such as miscarriage and compromised fetal development, pregnancy can trigger catastrophic APS (CAPS). Case reports have shown clinical benefit with eculizumab administration both in CAPS^181^, ^182^ and in a patient at high risk of developing CAPS,^183^ and a clinical trial is underway to evaluate eculizumab for CAPS following renal transplantation (NCT01029587). Together, animal studies and observations in women provide a strong case for a causative role for AP activation in adverse pregnancy outcomes in APS, and provide a strong rationale for clinical evaluation of AP inhibitors, at least in refractory APS if not in all APS pregnancies.

## 5 CONCLUSIONS

A successful pregnancy requires a measured amount of complement activation. Dysregulation of the AP undoubtedly contributes to the obstetric burden of adverse events in pregnancy. Events that take place in early pregnancy, detected by the AP activation fragment Bb, are predictive of adverse events arising at a later gestation. Dysregulated AP activation plays a clear role in APS‐related adverse obstetric events. Future research should focus on how we can harness this information to detect clinically‐quiescent disease before it leads to detrimental health effects to mother and infant. Identification of this subgroup of women in whom there is an established event in early pregnancy may inform treatment which, in future, might include complement‐directed therapeutics.

Research is also needed into the fourth trimester of pregnancy; the postpartum phase and beyond. The complications of pregnancy‐specific conditions dictate a woman's future health risk and exploring the role of the AP on postpartum vascular remodeling may provide insightful data relevant to attenuation of a woman's postobstetric cardiovascular risk, thus improving womens’ global health.

## CONFLICT OF INTEREST

KSJ none to declare. RAH is the owner and director of RAH Pharma Consulting Ltd. He also was employed by Novartis Pharma AG from 2001 to 2013, and between 2013 and 2022 held various consultancy agreements with Novartis.

## GLOSSARY

**Table:** 

Term	Definition
Zygote	Fertilized egg following union of a female ovum with a male sperm
Blastocyte	A group of dividing cells formed by a fertilized oocyte which then develops into an embryo
Decidua	Maternal component of maternal‐fetal interface. It only exists during pregnancy and comes from the endometrial lining of the uterus. Following labor, the decidua is shed, and only rebuilds if a subsequent pregnancy happens
Decidualization	Describes the functional and morphological changes that occur within the endometrium, enabling the blastocyte to implant and the placenta to develop
Extravillous cytotrophoblasts	The extravillous trophoblasts (EVT) depart from the tip of the placental villi and migrate into the maternal decidua, providing anchorage of the villi to the uterine wall. The EVT invade the spiral arteries (now termed endovascular trophoblast) and in union with the uNK remodel the spiral arteries
Placental villi	Houses the fetal blood vessels and comprises of the syncytiotrophoblast and cytotrophoblast layers. The villi are in direct contact with maternal blood enabling nutrient and gas exchange, they form “finger like” projections (chorionic villi)
Syncytiotrophoblasts	Outer syncytial layer of the trophoblast (fetal origin) that invades uterine wall forming outermost fetal component of placenta. Formed from the fusion of two or more of the underlying cytotrophoblasts (hence multinucleated cells). The syncytiotrophoblasts are the major cellular barrier between the fetal compartment and maternal blood
Cytotrophoblasts	Reside directly underneath the syncytiotrophoblasts. They are undifferentiated mononucleated cells in the chorionic villi and differentiate into either syncytiotrophoblasts or extravillous cytotrophoblasts
Semi‐allogenic feto‐placental unit	The placenta and developing fetus as a unit, deriving, half of its genetics from the mother and the other half inherited from the father
Uterine natural killer cells	Natural killer cells within the decidua that have a distinct phenotype compared to peripheral blood natural killer cells
Zona pellucida	The extracellular matrix surrounding the blastomere membrane
Decidual stromal cells	The main cellular component of maternal decidual layer
Haemochoiral placenta	A placenta where maternal blood comes into direct contact with the fetal chorion (the outermost fetal membrane)
Pre‐eclampsia	Pregnancy‐specific condition defined by hypertension with accompanying maternal organ dysfunction
HELLP syndrome	A pregnancy‐specific condition characterized with Hemolysis, elevated liver enzyme and low platelets, considered a severe variant of pre‐eclampsia
Preterm birth	Birth of neonate between 20^0/7^–36^+6/7^ weeks' gestation

## Data Availability

Data sharing not applicable to this article as no datasets were generated or analysed during the current study.
